# New Metallic Ordered Phase of Perovskite CsPbI_3_ under Pressure

**DOI:** 10.1002/advs.201900399

**Published:** 2019-05-20

**Authors:** Yongfu Liang, Xiaoli Huang, Yanping Huang, Xin Wang, Fangfei Li, Youchun Wang, Fubo Tian, Bingbing Liu, Ze Xiang Shen, Tian Cui

**Affiliations:** ^1^ State Key Laboratory of Superhard Materials College of Physics Jilin University Changchun 130012 P. R. China; ^2^ Division of Physics and Applied Physics School of Physical and Mathematical Sciences Nangyang Technological University 637371 Singapore; ^3^ Centre for Disruptive Photonic Technologies The Photonics Institute Nanyang Technological University 637371 Singapore

**Keywords:** electronic structure, high pressure, metallization, perovskites, phase transition

## Abstract

Pressure‐induced electronic structure transition from insulating phase to metal state is a potential new paradigm for halide perovskites. The metallization based on these materials may afford a novel motif toward realizing new electronic properties even superconductivity phenomenon. Herein, how static compression modulates the crystal and electronic structure of typical perovskite semiconductors cesium lead iodine (CsPbI_3_) by both experimental and theoretical studies is reported. The comprehensive studies discover the insulator–metal transition of CsPbI_3_ at 39.3 GPa, and reveal the key information behind the electronic transition. The perovskite's precise structural evolution is tracked upon compression, from orthorhombic *Pnma* phase to monoclinic *C2/m* structure before the metallic transition. More interestingly, the *C2/m* phase has the most distorted octahedra and the shortest Pb–I bond length relative to the average bond length that is ever reported in a halide perovskite structure. The electronic transition stems from the structural changes accompanied by the anomalously self‐distorted octahedra. These studies show that pressure can significantly alter the structural and electronic properties of these technologically important perovskites.

Metal halide perovskites (MHPs) are currently under intensive focus due to their unique chemical compositions, crystal structures, superior magnetic^1^ and optoelectronic properties,^2^ which have been proposed for multifarious applications such as light‐controlling magnetic devices,[qv: 1b] light‐emitting diodes,^3^ photovoltaics,^4^ and photonics.^5^ Perovskite compounds have a common chemical formula ABX_3_, and its structure can be alternatively viewed as corner‐linked BX_6_ octahedra with interstitial A cations. The structural transition of MHPs is a crucial one that affects the stability of perovskite materials and has received a growing number of attentions. Pressure also provides a new route to modify the crystal structures of these materials thus tuning the corresponding optical and electrical properties.^6^

Along with the structural transitions, the application of pressure is also able to modify the local electronic Fermi surface structure between conduction and valence band of MHPs and narrow conversion of the Fermi level where further metallization ensues in certain MHP species. Recently, Jaffe et al. found that the conductivity of copper‐chloride hybrid perovskite increased by five orders of magnitude up to 50 GPa and also observed the metallization of methylammonium lead iodine at 62 GPa by resistance measurements and IR reflectivity experiments.[qv: 6a,b] Up to now, the reported metallization in MHPs merely initiated from long‐range ordered crystal structure pulverization, i.e., amorphization, yielding obliterated the periodic structure. For example, α‐formamidinium lead iodine became amorphous above 4.5 GPa, which may predominate in the metallization at 53 GPa.[qv: 6c] In principle, the BX_6_ octahedra are usually distorted, or tilted and rotated relative to neighboring octahedra by modifying bond length and bond angle during the structural transitions in perovskites. However, how pressure modulating the crystal and electronic structures remains elusive, and the relationship between structural and electronic transition is also unambiguous, especially the role of octahedral configuration during these transitions.

As a promising inorganic hybrid perovskite, cesium lead iodine (CsPbI_3_) possesses much better phase stability through bifunctional gradient halide doping and organic cation surface passivation,^7^ and is much more superior in photo‐ and thermal‐stability, even moisture‐resistant after the formation of a organic cation terminated surface.^8^ Although several studies have investigated pressure‐induced structure transition of CsPbI_3_, they are limited in relatively low‐pressure regimes (below 15 GPa) and the unambiguous determination of crystal symmetry even the refinement of atomic fractional coordinate are challenging.^9^ Herein, we have tracked CsPbI_3_ perovskite as a typical example to investigate both structural and electronic changes at high pressure and unearth the inherent mechanism, by the joint of experimental and theoretical studies. Both infrared reflectivity and temperature‐dependent electrical resistivity demonstrate that pressure strongly modulates its band structure from insulating to metallic. Along with the pressure‐induced metallization of CsPbI_3_, the structural evolution has been determined especially the anomalously self‐distortion of octahedral configurations.

After loading the target sample into the diamond anvil cell (DAC), it displays piezochromic transitions from translucent yellow to opaque black upon compression (**Figure** **1**a). The measured corresponding optical absorption and bandgap under pressure are shown in Figure 1b,c. We have observed that the bandgap of CsPbI_3_ experiences a largely redshift from 2.5 to 1.2 eV as pressure increased to 15 GPa, firstly reaching the Shockley–Queisser optimum bandgap (1.34 eV) in inorganic hybrid perovskites, at which the maximum theoretical energy‐conversion efficiency of solar cells is 33%.^10^

**Figure 1:**
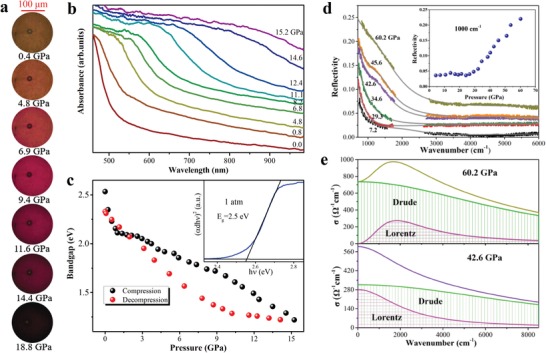
a) Optical images of CsPbI_3_ in a diamond anvil cell (DAC) upon compression in the pressure range 0.4–18.8 GPa. b) The selected optical absorption spectra as a function of pressure during compression. c) Evolution of bandgap under pressure. The illustration shows selected bandgap Tauc plots for CsPbI_3_ at 1 atm. d) The infrared reflectivity in the mid‐infrared spectral range of CsPbI_3_ at pressures up to 60.2 GPa. The solid lines represent Lorentz–Drude (LD) model fits to the data. The small gap in the spectrum (1700–2600 cm^−1^) is due to diamond anvil absorption. The inset displays the pressure dependence of the reflection at 1000 cm^−1^. e) Real part σ_1_(ω) of the complex optical conductivity achieved from the selected spectrum of 42.6 and 60.2 GPa in panel (d), and the fit components containing Drude and Lorentz parts.

These phenomena indicate that CsPbI_3_ undergoes a large electronic evolution where bandgap narrowing is followed by possible metallization transition. We therefore obtained high‐pressure reflectivity spectra of CsPbI_3_ from 800 to 6000 cm^−1^ in Figure 1d. With increasing pressure, we observe a Drude‐like mode, contributed from free charge carriers,^11^ showing sharply increasing reflectivity with decreasing frequency, in contrast to a spectrum recorded at 7.2 GPa. This indicates the transition of CsPbI_3_ to a metallic state. The inset in Figure 1d displays the pressure dependence of the reflectivity at 1000 cm^−1^, showing a drastic increase in reflectivity started above 29.3 GPa. With further compression to 60.2 GPa, the reflectivity increased up to 0.22, the highest one we have measured. A significant increase in reflectivity takes place on a low energy side, and shifts to high energies with increasing pressure, as expected in a free electron metal.

The reflectivity results provide spectroscopic evidence for the pressure‐induced metallization in CsPbI_3_. For the analysis of reflectivity, the measured *R*_s–d_ on CsPbI_3_ needs first to be interpolated within the diamond absorption range (1700–2600 cm^−1^). Figure 1d highlights the undertaken steps to achieve this goal. Then we fit our reflectance spectrum within the Lorentz–Drude phenomenological approach by using RefFIT.^12^ It is consisted of fitting the dielectric function by the following expression

(1) $$\tilde{\varepsilon }\left(\omega \right)=\varepsilon_{1}\left(\omega \right)+i\varepsilon_{2}=\varepsilon_{\infty }-\frac{\omega_{p}^{2}}{\omega^{2}+i\omega \gamma_{D}}+\sum_{j}\frac{S_{j}^{2}}{\omega_{j}^{2}-\omega^{2}-i\omega \gamma_{j}}$$

where ε_1_ and ε_2_ are the real and imaginary parts of the dielectric function, respectively. ε_∞_ is the so‐called “high‐frequency dielectric constant,” which represents the contribution of all oscillators at very high frequencies. ω_p_ and γ_D_ are the plasma frequency and the width of the Drude peak, whereas *ω_ϳ_*, *γ_ϳ_*, and *S_j_* ^2^ are the center‐peak frequency, the width, and the mode strength for the *j*th Lorentz harmonic oscillator (HO), respectively. The knowledge of $\tilde{\varepsilon }$(ω) gives us the access to all optical functions and finally allows us to reproduce the measured *R*_s–d_(ω) spectra. The optical properties of CsPbI_3_ at various pressures are well described by one Drude term for metallic component and one Lorentz term of HO for lattice vibrations or the interband transitions.^11^, ^13^ The fit quality over the entire spectral range is remarkably good for our sample (see Figure 1d). The corresponding optical conductivity σ_1_(ω) obtained through standard Kramers–Kronig transformation,^14^ is displayed in Figure 1e. At 42.6 GPa, the rapidly increased σ_1_(ω) is dominated by the Drude peak, indicating that the sample transforms into a metallic state. Up to 60.2 GPa, the σ_1_(ω) demonstrates an enhancement of the metallicity in the target sample.

We further carried out electrical resistivity studies to confirm the pressure‐induced metallization of CsPbI_3_ up to 60 GPa. Electrical resistivity is one of the most important parameters for photovoltaic materials. **Figure** **2**a shows the electrical resistivity changes of CsPbI_3_ in the pressure range of 25–60 GPa at room temperature. For CsPbI_3_, the decrease of resistivity is nearly exponential, fit by log (ρ) = 2.3(2)–0.096(4) *P*, resistivity (ρ) in unit of Ω cm^−1^ and pressure (*P*) in unit of GPa. The electrical resistivity of CsPbI_3_ continuously decreases more than four orders of magnitude from 25.7 to 62.1 GPa. Above 27.6 GPa, variable‐temperature dc Four‐point probe electrical technique was employed to detect the resistance change as a function of pressure. We have obtained the temperature–pressure–resistivity contour map in Figure 2b, clearly showing the region between semiconductor and metal. The temperature (*T*)–resistivity (ρ) exhibits negative dρ/d*T* at 27.6 GPa (see Figure 2c), and this means the presence of a semiconducting state owing to thermally activated carriers. Above 34.3 GPa, positive dρ/d*T* is observed in all measured temperatures, which is a hallmark of metallic character.

**Figure 2:**
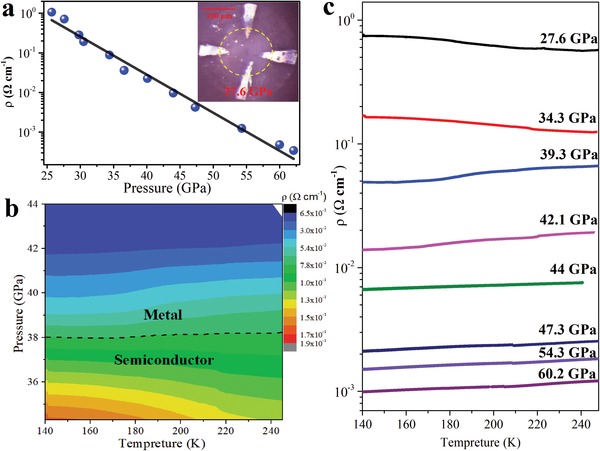
a) Room temperature electrical resistivity at pressure, and the line shows a linear fitting of log ρ versus pressure (equivalent for an exponential fitting of ρ vs pressure). The inset is the optical image of a DAC containing CsPbI_3_ contacted by four platinum leads. b) Temperature–pressure–resistivity contour map. c) The pressure dependence of resistivity in CsPbI_3_ as a function of temperature, upon compression to the highest pressure of 60.2 GPa.

We subsequently investigated the crystal structures under high pressure to acquire structural information concerning the microscopic mechanisms of the insulator–metal transition. In situ high‐pressure synchrotron XRD measurements were carried out up to 64 GPa and the representative diffraction patterns at selected pressures are shown in Figure S1a of the Supporting Information. At 0.1 GPa, CsPbI_3_ crystallizes into the orthorhombic *Pnma* phase with one‐dimensional double chains of edge‐sharing PbI_6_ octahedra, and Cs atoms that are ninefold coordinated by I atoms. The refined lattice constants of *Pnma* phase are *a* = 10.458 (3) Å, *b* = 4.815 (2) Å, and *c* = 17.776 (5) Å (see **Figure** **3**a), in agreement with the previously reported results.^15^ With increasing pressure, the systematic increase in the Bragg peaks to higher diffraction angle (2θ) is consistent with the contraction of the unit cell (Figure S1b, Supporting Information). The appearance of the new Bragg peaks in the XRD patterns indicates that a first order‐like structural transition has taken place, while the high pressure and ambient pressure phase coexist in the pressure range of 6.9–18 GPa. Upon further compression, the high‐pressure phase sustains up to 64 GPa without further structural transitions.

**Figure 3:**
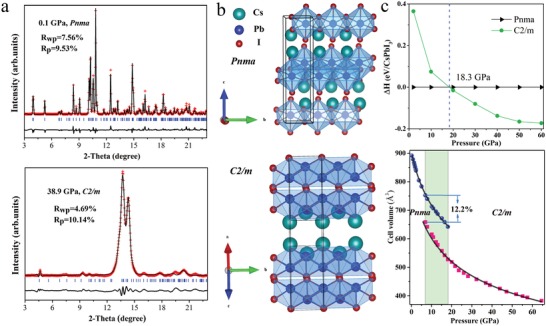
a) Rietveld refinement of XRD patterns at 0.1 and 38.9 GPa (λ = 0.6199 Å). The solid symbols and lines represent the experimental and calculated data, respectively, and the solid lines at the bottom are the residual intensities. The vertical bars indicate the peak positions. b) Crystal structures of CsPbI_3_ with symmetry *Pnma* and *C2/m*. c) Pressure dependence of the enthalpy difference and cell volume. The solid lines are the Birch–Murnaghan fitting curves to the experimental *V*(*P*) data.

To characterize the high pressure crystal structure and the mechanism of the phase transitions, complementary first‐principles calculations were needed. We have performed structural searches for stable compounds by using a variable‐composition evolutionary structure prediction algorithm, as implemented in the USPEX code.^16^ We have found a new phase with lower enthalpy than the ambient *Pnma* phase at pressures above 18 GPa (Figure 3c), in line with the experimental transition pressure. The new phase is identified as a layered monoclinic *C2/m* structure and its polyhedra reveal sandwiches stacking order similar to *Pnma* phase. But the octahedra in the *C2/m* phase are greatly distorted in stark contrast to those in the *Pnma* phase. Phonon calculations clearly indicate dynamical stability of *C2/m* structure above 18 GPa (Figure S2, Supporting Information).

Details of the structural refinement of the high‐pressure structures are presented in Figure 3a. At 38.9 GPa, the XRD profile is well reproduced by the *C2/m* structure with the refined lattice parameters *a* = 15.423 (2) Å, *b* = 3.511 (3) Å, *c* = 18.461 (1) Å, and β = 132.829 (2)°. Figure 3c shows the obtained equation of state (EOS) together with the phase diagram, where only the compressional data are used for third‐order Birch–Murnaghan EOS fitting. For *Pnma* and *C2/m* phase, the fitting yields bulk moduli and cell volumes of *B*_0_ = 27.7 (2) GPa, *V*_0_ = 900.4 (4) Å^3^, and *B*_0_ = 18.6 (3) GPa, *V*_0_ = 807.9 (2) Å^3^. A large structural reconstruction is observed during the first‐order transition structural transition accompanied with 12.2% volume drop. The behavior of CsPbI_3_ under high pressure offers a case where the perovskite structure can be densified by significantly shortening the A–B distance (chemical formula ABX_3_) and distorting the octahedra.

To better understand the electronic structure evolution of CsPbI_3_ that determines their highly tunable optical and electrical transport properties, we have performed the band structure, total density, and projected density of states (DOS) calculations for CsPbI_3_, as presented in **Figure** **4**. For the *Pnma* phase at 2 GPa, the calculated indirect fundamental bandgap is 1.88 eV in agreement with our experimental value. The bandgap is remarkably reduced when the pressure increased to 10 GPa. Upon compression to 25 GPa, the structural transition has finished as discussed above, and the monoclinic *C2/m* phase with indirect bandgap of 0.49 eV occurs. At 50 GPa, the bandgap is closed (Figure S5, Supporting Information). In addition, the nonmetallic character of *C2/m* structure disappears, and the metallic character appears indicated by the conduction band along the *G* and *V* symmetry line through the Fermi level at 60 GPa. Meanwhile, Figure 4 also presented the DOS projected on the atomic orbitals of the Cs, Pb, and I atoms in the *Pnma* and *C2/m* phases from our calculations. At 2 GPa, the DOS for the *Pnma* phase shows that the valence band maximum (VBM) is virtually attributed to the interactions between I 5p and Pb 6s orbitals in the [PbI_6_]^4−^ octahedral network, whereas the conduction band minimum (CBM) was determined by antibonding hybridization between Pb 6p and I 5p. The contribution of Cs^+^ cation to VBM and CBM is negligible. Note that the VBM state has almost a I 5p character, owing to the higher energy level and much more electrons of the I 5p orbital compared with that of the Pb 6s state. The CBM was almost totally contributed from Pb 6p state, because Pb 6p orbital possesses a much greater energy level than I 5s state. With continuous compression to 10 GPa, I 5s obtains growing energy, finally the CBM is dominated by Pb 6p and I 5s. The DOS analysis also shows some distinctly different features between the *Pnma* and the *C2/m* phases. In the *C2/m* case, Figure 4c, the contribution of I 5p orbitals in the CBM is noticeably enhanced. Up to 50 GPa, a remarkable transition from insulator to metal emerges accompanied with the overlap between I 5p and Pb 6p orbital (Figure S5, Supporting Information). At the Fermi level, I 5p state in the *C2/m* structure dominates mostly, which is probably largely contributed by the metallic element iodine. The element iodine is reported to be a metal at about 16 GPa.^17^ Its pressure‐induced gradual metallization has been explained by the continuous increase in the band overlap between the valence and the conduction band. In this respect, we can infer that the analogues compounds CsPbBr_3_ and CsPbCl_3_ have not been found the similar metallic transition, possibly related with the corresponding halogen elements with much higher metallic pressure than element iodine.

**Figure 4:**
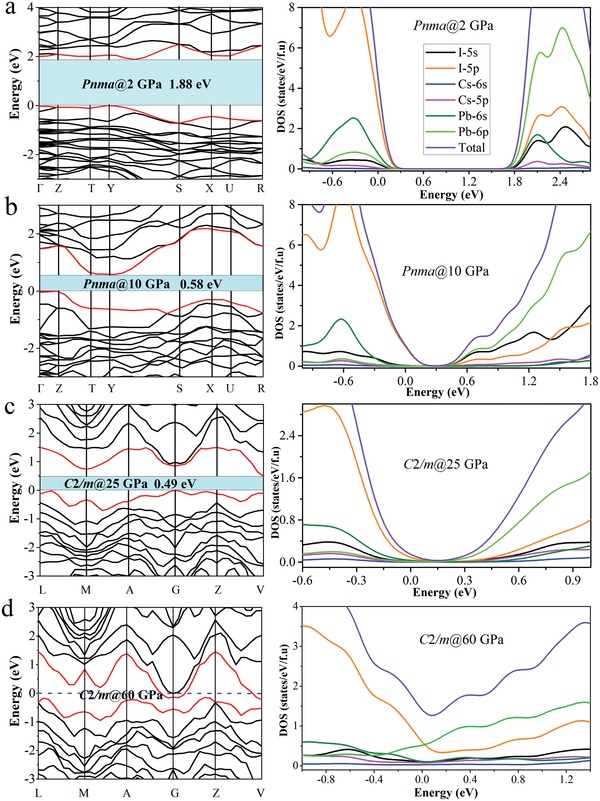
The band structures and DOS at a) 2 GPa, b) 10 GPa, c) 25 GPa, and d) 60 GPa, respectively. Blue dotted line shows the Fermi level (*E*_F_).

The emergence of metallic CsPbI_3_ perovskite can be understood in the framework of octahedral polymorph and electron localization function (ELF) described below. The application of pressure reduces interatomic distances and profoundly modifies electronic orbitals and bonding patterns. In this work, with increasing pressure, the unique variation for the octahedra has been found during the structure transformation from orthorhombic *Pnma* into monoclinic *C2/m* phase, which is shown in **Figure** **5**a,d. Before the structural transition, both Pb–I bond lengths and Pb–I–Pb bond angles are anomaly shortened when the perovskite lattice is compressed to 10 GPa (Figure 5b,c). Simultaneously, the enhanced orbital coupling between the Pb 6p and I 5p states (Figure S7a, Supporting Information) pushed down the CBM due to its antibonding character, which explains the sharp decrease of the bandgap with pressure at low pressure. At 10 GPa, the Pb–I bond lengths (except Pb‐I4) show sudden lengthening and Pb–I–Pb bond angles are also enlarged, and three abnormal Pb–I stretching modes are detected by the Raman spectra experiment shown in lilac area of Figure S6b of the Supporting Information. And CBM happened to be dominated by the Pb 6p and I 5s orbitals, as displayed in Figure 4b. Accordingly, the bandgap narrowing can be interpreted from the significant strong coupling of the Pb 6p and I 5s orbitals, which caused by the enlarged Pb–I–Pb bond angles and shortened Pb‐I4 shown in Figure 5c and Figure S7b (Supporting Information). When a much higher pressure was applied, a phase transition from orthorhombic to monoclinic structure occurred. The [PbI_6_]^4−^ octahedra underwent a stark distortion to accommodate the Jahn–Teller effect, and the averaged Pb–I–Pb bond angles and Pb–I bond lengths markedly decreased in monoclinic phase with increasing pressure (Figure 5b,c). The reduction of bond lengths played a dominant role in the decrease of the bandgap energy,^18^ which resulting in the increased overlap between electron clouds of CBM dominated Pb 6p and I 5p (Figure S7c, Supporting Information), and VBM dominated Pb 6s and I 5p. Its bandgap induces large movements of the orbitals toward the *E*_F_. Thereby increasing electronic band dispersion accompanied by pushing up the VBM and pulling down the CBM, as eventually reflected in bandgap overlap in energy range, and metallic band structures are obtained. And the calculated ELF in Figure 5d also shows the similar changes from 2 to 50 GPa. Comparing with the null charge distribution between two neighboring octahedra at 2 GPa, the electron distributions gradually diffused at high pressure, with the decrease of the Pb–I distance. These findings provide consistent evidence with pressure‐induced variation of crystal structure (i.e., volume contraction and distortion of PbI_6_ octahedral units), resulting in the changes of electronic structure.

**Figure 5:**
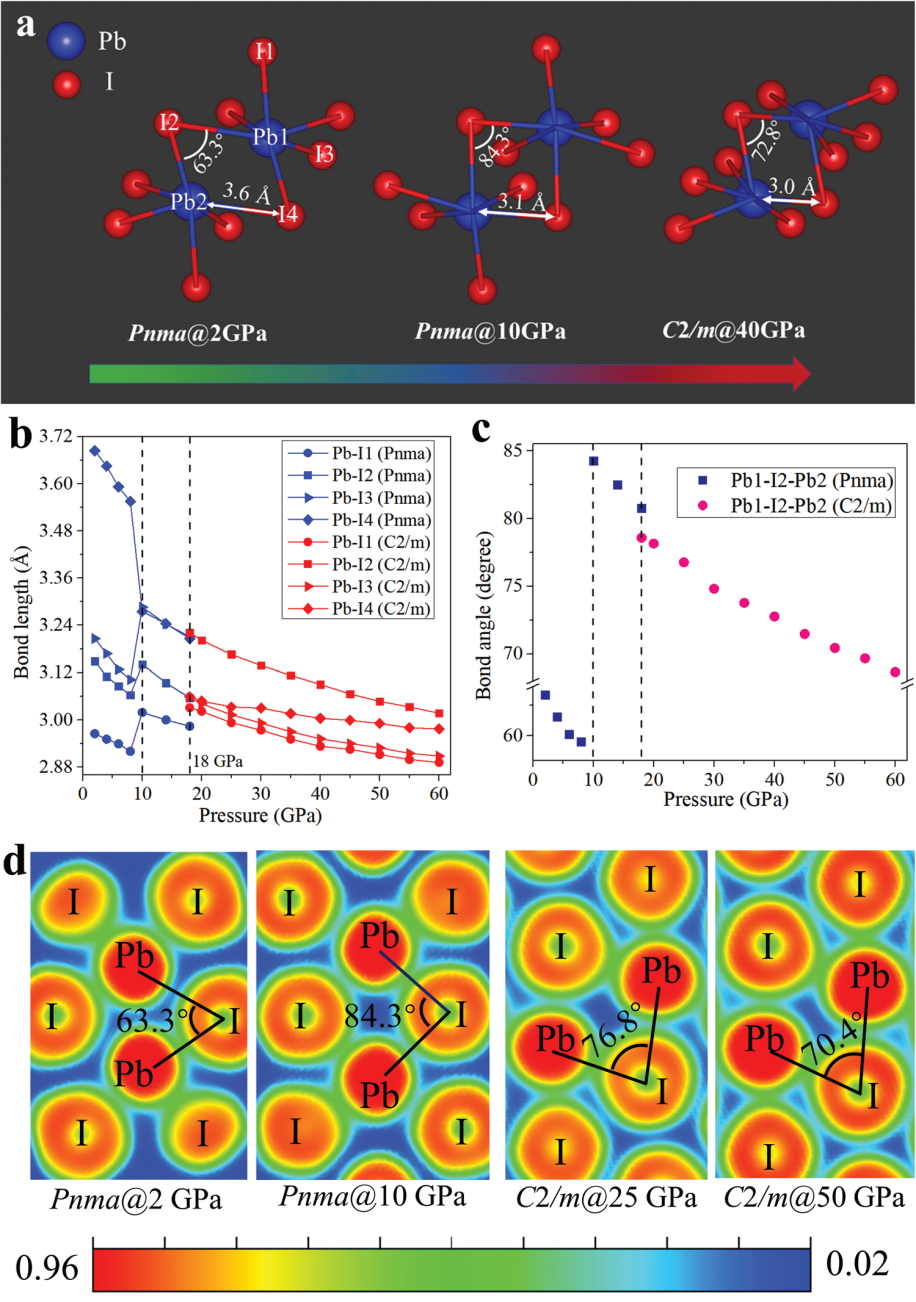
a) The octahedra illustrations of orthorhombic and monoclinic CsPbI_3_ perovskites under high pressure. The calculated b) bond length of Pb–I and c) bond angle of Pb–I–Pb with increasing pressure. d) The calculated electron localization function (ELF) of CsPbI_3_ under pressure. ELF of *Pnma* phase in the (212) plane at 2 and 10 GPa, and *C2/m* phase in the (−1 3 −13) plane at 25 and 50 GPa.

In summary, we comprehensively investigated the electronic and structural evolutions of CsPbI_3_ under high pressure by combining the experimental measurements and theoretical calculations. The precise structural evolution of CsPbI_3_ upon compression is discovered, from *Pnma* phase to *C2/m* structure at 6.9 GPa through a mixed region, accompanied by the changes of octahedra. More interestingly, *C2/m* phase has the most distorted octahedra and a shortest Pb–I bond length relative to the average bond length that has ever been reported in a halide perovskite structure. Our comprehensive experiments discover the insulator–metal transition of CsPbI_3_ at 39.3 GPa because of the anomalously distortion in octahedra, also validated by the calculation. Therefore, application of pressure provides an additional tuning knob for accessing further electronic and optical diversity from this exceptionally versatile family of materials.

## Conflict of Interest

The authors declare no conflict of interest.

## Supporting information

Supplementary
