# Supplementary material for: New Metallic Ordered Phase of Perovskite CsPbI3 under Pressure
Source: Adv Sci (Weinh). 2019 May 20;6(14):1900399. doi: 10.1002/advs.201900399 (PMC6661939; doi:10.1002/advs.201900399)
Supplement: Supplementary file 1 — Supplementary [file ADVS-6-1900399-s001.pdf]

Copyright WILEY-VCH Verlag GmbH & Co. KGaA, 69469 Weinheim, Germany, 2019.

Supporting Information

for *Adv. Sci.,* DOI: 10.1002/advs.201900399

New Metallic Ordered Phase of Perovskite $CsPbI_3$ under Pressure

Yongfu Liang, Xiaoli Huang, Yanping Huang, Xin Wang, Fangfei Li, Youchun Wang, Fubo Tian,* Bingbing Liu, Ze Xiang Shen, and Tian Cui**

Copyright WILEY-VCH Verlag GmbH & Co. KGaA, 69469 Weinheim, Germany, 2019.

Supporting Information

New metallic ordered phase of perovskite $CsPbI_3$ under pressure

Yongfu Liang[1], Xiaoli Huang[1,], Yanping Huang[1], Xin Wang[1], Fangfei Li[1], Youchun Wang[1], Fubo Tian[1,*], Bingbing Liu[1], Ze Xiang Shen[2,3] and Tian Cui[1,*]*

[1]State Key Laboratory of Superhard Materials, College of Physics, Jilin University, Changchun 130012, P.R. China.

[2]Division of Physics and Applied Physics, School of Physical & Mathematical Sciences, Nanyang Technological University, Singapore.

[3]Centre for Disruptive Photonic Technologies, The Photonics Institute, Nanyang Technological University, Singapore.

*Corresponding author, E-mail: huangxiaoli@jlu.edu.cn, tianfb@jlu.edu.cn, and cuitian@jlu.edu.cn

Experimental Section

In Situ High-Pressure IR reflectivity Experiments. Cesium lead iodine with purity of >99% was purchased from Xi'an Polymer Light Technology Corp. The symmetry-type diamond anvil cell (DAC) was employed to generate high pressure. IIa-type ultralow fluorescence diamonds with culet size of 200 μm were used for high pressure IR reflectivity experiments. One sheet of T301 stainless steel gasket was pre-indented to 45 μm in thickness with a hole of 80 μm in diameter was drilled in the center of the indentation by laser to serve as sample chamber. The ruby fluorescence method was used for the pressure calibration.[1]

The IR reflectivity measurements were conducted at room temperature using a Bruker Vertex 80 V FT-IR spectrometer (BRUKER OPTIK GMBH, Germany) in the range of 600–8000 cm^{-1} equipped with a nitrogen-cooled mercury-cadmium-telluride (MCT) detector. The $CsPbI_3$ was directly contacted to culet surface of the diamond anvil, and no medium was used. Pressure-induced reflection spectra were measured at the interface between the sample and diamond anvil. To normalize the sample spectra, the spectra reflected from the inner diamond−air interface of an empty cell served as the reference.[2] The obtained reflectivity spectra $R_{s-d}(\omega)$refer to the absolute reflectivity at the sample-diamond interface calculated by

$$R_{s-d}(\omega) = \frac{I_{s-d}(\omega)}{I_d(\omega)} \cdot \frac{I_d(\omega)}{I_e(\omega)} \cdot \frac{I_d(\omega)}{I_0(\omega)}, \quad (2)$$

Where I_{s-d} is the intensity reflected from the sample-diamond interface, $I_d(\omega)$ is the power reflected off of the air-diamond interface (the back plate of diamond), $I_e(\omega)$is the power reflected from the empty cell (through the diamond culet), and I_0 is the

power reflected from a polished gold mirror (taken as a perfectly reflecting reference surface). The ratio I_d/I_0 is taken to be 0.18.[3]

In Situ High-Pressure XRD Experiments. Ia-type diamonds with culet size of 200 μm were used for high pressure XRD experiments. One sheet of T301 stainless steel gasket was pre-indented to 45 μm in thickness with a hole of 80 μm in diameter was drilled in the center of the indentation by laser to serve as sample chamber. The ruby fluorescence method was used for the pressure calibration[1]. In situ high-pressure XRD patterns were collected at the beamline 4W2 of Beijing Synchrotron Radiation Facility(BSRF) and 15U1 of Shanghai Synchrotron Radiation Facility (SSRF) using angle-dispersive XRD source (λ = 0.6199 Å). CeO_2 was used as the standard sample to do the calibration. And the transition into standard one-dimensional powder patterns versus 2θ was carried out using the FIT2D software. Materials Studio Software was employed to index and refine the collected experimental XRD profiles.

In situ conductivity measurements. The diamond anvil cell (DAC) made of BeCu alloy with culet of 300 μm in diameter was utilized. A T301 stainless steel was pre-indented into 45 μm in thickness, and then a hole of 140 μm in diameter was drilled at the center of indentation. Then, a mixture of cubic BN and epoxy was compressed into the indentation was used as the insulating layer. Subsequently, another hole of 120 μm was drilled to serve as a sample chamber. Solid $CsPbI_3$ was loaded into the chamber onto which four platinum leads were overlaid. A constant current of 1 mA was passed between two adjacent leads while voltage was measured

across the other two according to the van der Pauw method. Two resistance values are required to calculate sheet resistance R_s: a horizontal resistance R_h found by measuring voltage across the first and second leads, then a vertical resistance R_v from the voltage across the second and third leads, for example. Here, R_s (Ω) is given by the following equation:

$$e^{-\pi R_h/R_s}+e^{-\pi R_v/R_s}=1 \quad (3)$$

From R_s, one can then calculate resistivity, ρ (Ω·cm), by multiplying R_s by the thickness, t (cm).[4], [5] For temperature-dependent four-probe resistivity measurement. The temperature was cooled by the Janis PTSHI-950 systems, and detected by the model 26 cryogenic temperature controller. A constant current of 1mA was passed between two adjacent leads while voltage was measured across the other two.

Theoretical Section

First-Principles Calculations. We have investigated the high pressure structures of $CsPbI_3$ by combining density functional theory (DFT) and ab initio evolutionary algorithm USPEX.[6] The pressures of structure search were set at 30, 40, and 60 GPa and the simulation cells containing up to eight units. All structural optimizations, electronic calculations, and phonon spectrum were performed using the CASTEP code.[7] The local density approximation (LDA-CAPZ)[8] approach of exchange-correlation functional was employed with the plane-wave energy cutoff 910 eV and the k-point spacing $2\pi\times 0.03$ Å^{-1} in the Brillouin zone.

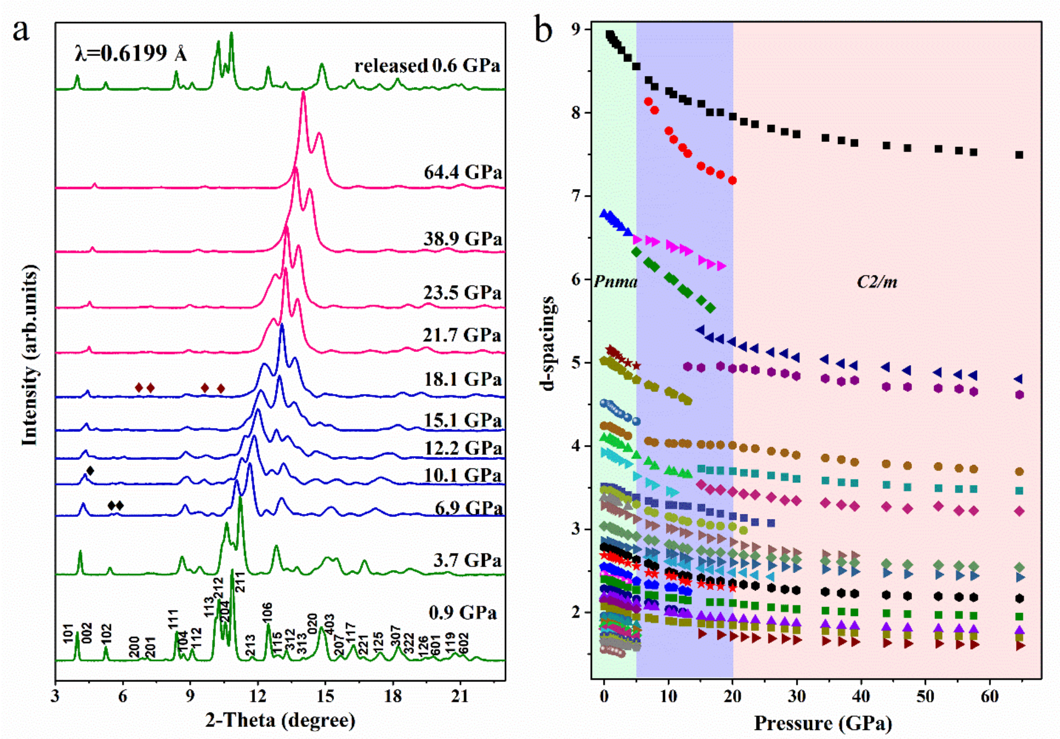

Figure S1. Representative synchrotron X-ray diffraction (XRD) patterns of $CsPbI_3$ under high pressure. (a)The selected XRD patterns of $CsPbI_3$ upon compression and decompression. (b) The *d*-spacings of the diffraction peaks as a function of pressure.

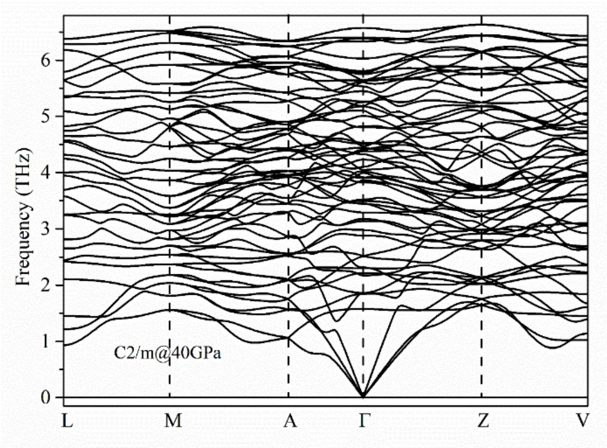

Figure S2. The calculated phonon spectra of *C*2/*m* at 40 GPa.

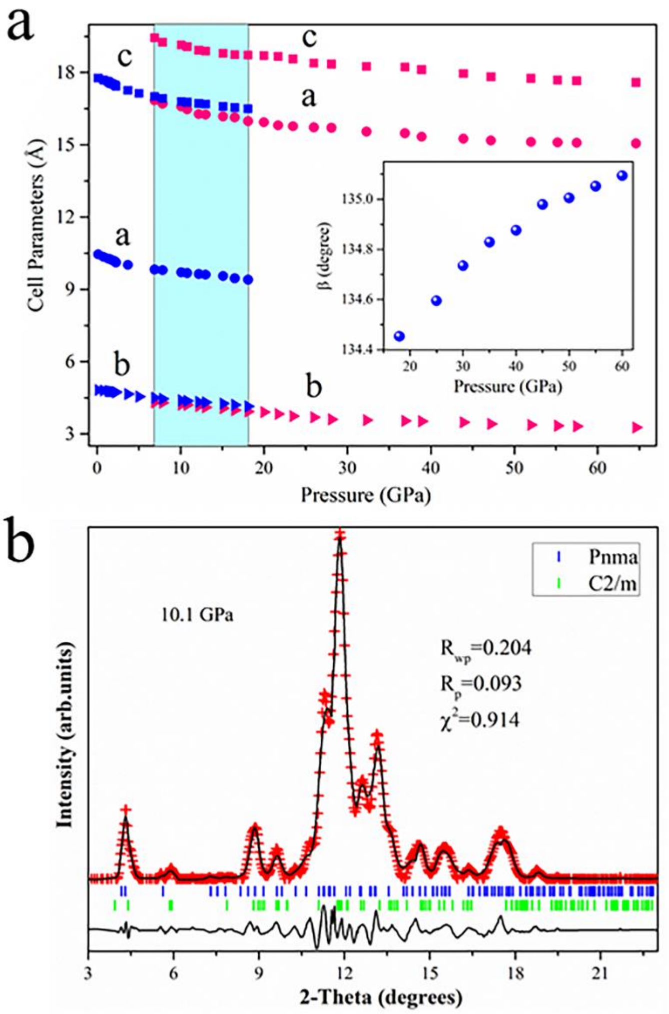

Figure S3. a) Lattice parameters evolution at various pressure, and the inset picture shows β at different pressure. b) Rietveld refinement of XRD patterns at 0.1 GPa.

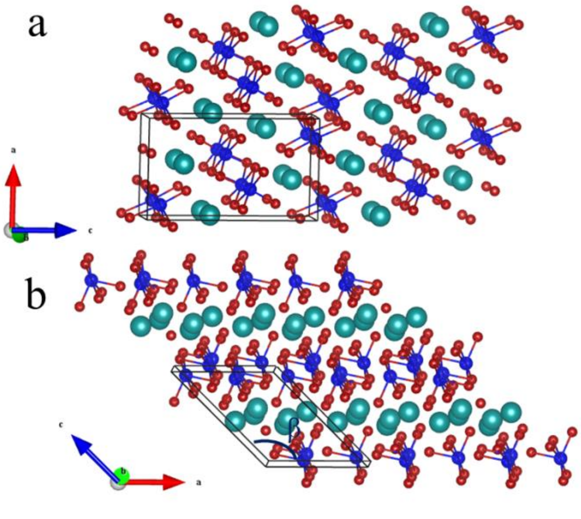

Figure S4. The crystal structures of $CsPbI_3$. (a) Ambient pressure *Pnma* phase, (b) high-pressure *C2/m* phase.

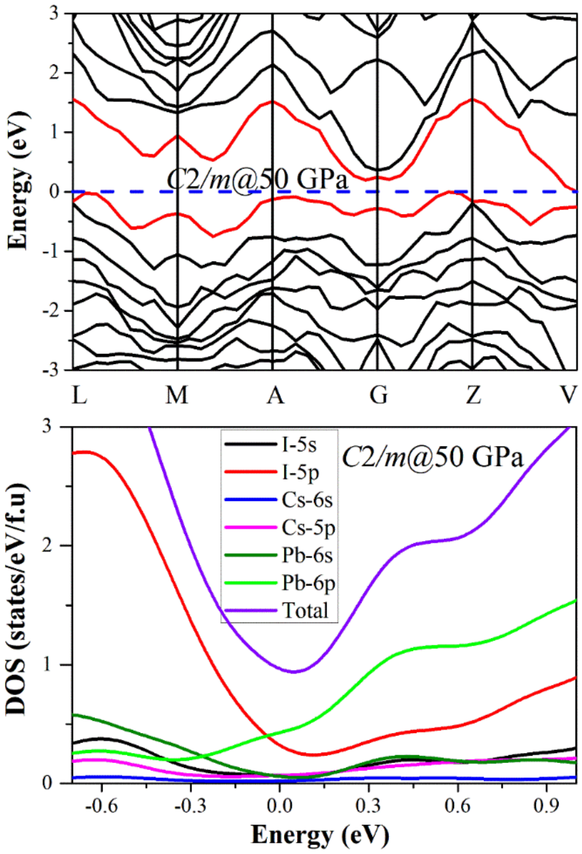

Figure S5. The calculated electronic band structure and projected density of states (DOS) of $CsPbI_3$ at 50 GPa.

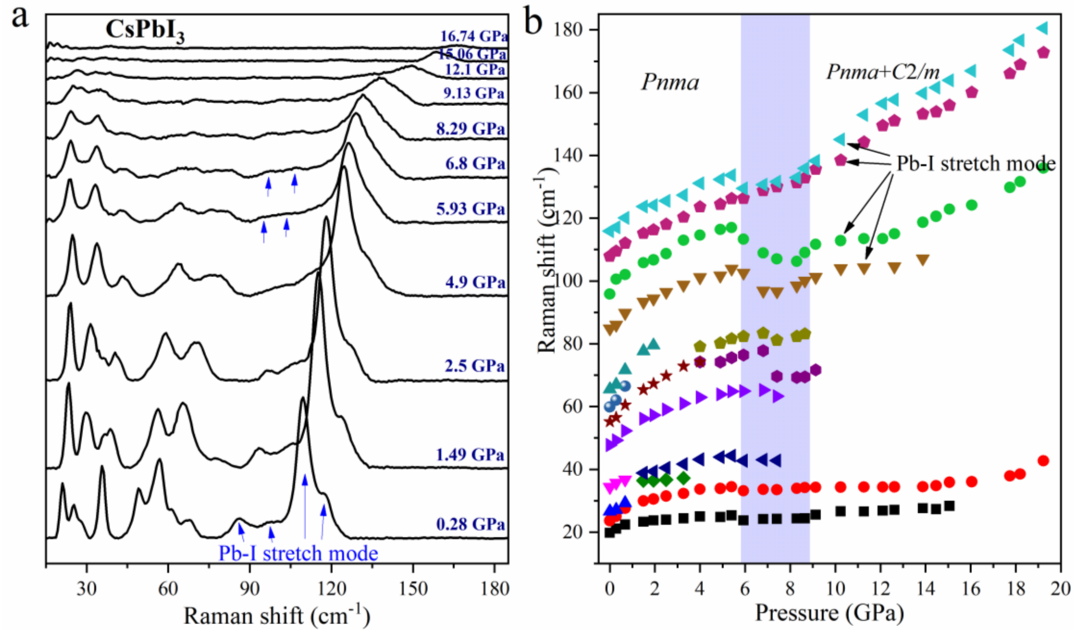

Figure S6. The Raman spectra and vibration modes upon compression to 16.7 GPa. (a) The selected Raman spectra at different pressures. (b) The evolution of Raman vibration modes as a function of pressure.

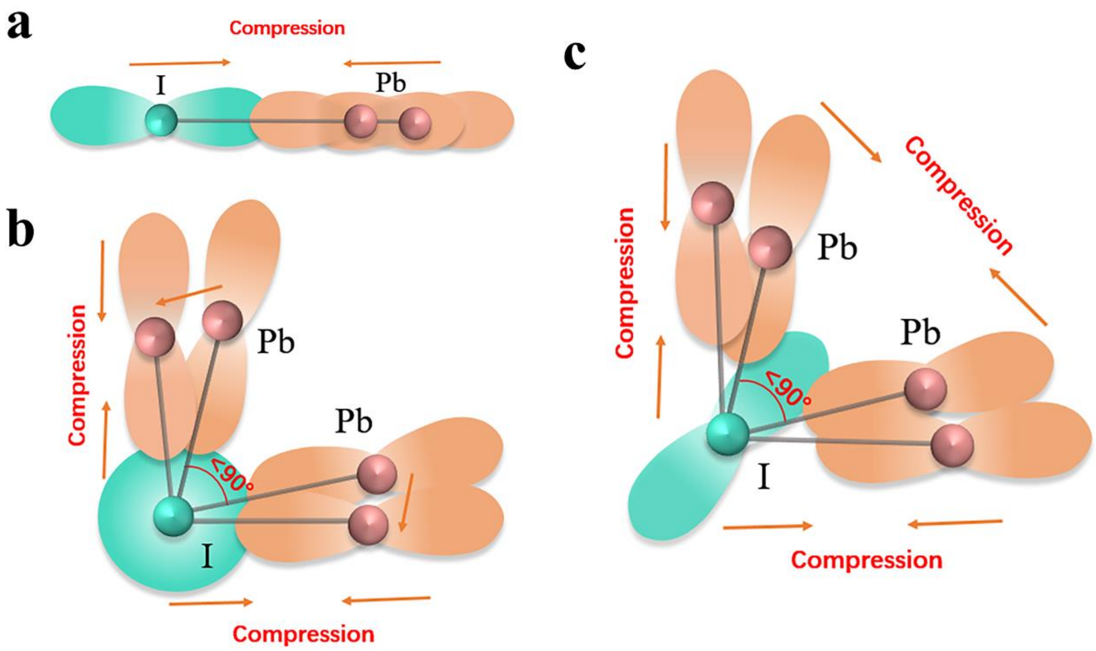

Figure S7. The schematic models of the Pb-I bond length and Pb-I-Pb bond angle. **a**) The enhanced orbital coupling between the Pb 6*p* and I 5*p* states. Model [b)] represent Pb-I4-Pb, the enlarged bond angle and shortened bond lengths enhanced the coupling of Pb 6*p* and I 5*s* orbitals. c) The enhanced orbital coupling between Pb 6*p* and I 5*p* with shortened bond lengths and decreased bond angle.

Table S1. The refined lattice parameters and atomic positions of *C*2/*m* structure in $CsPbI_3$ at 40 GPa

**Space group**	*C*2/*m*		**Volume (Å^3)**		437.99	
**Cell parameter (Å)**	***a***		***b***	***c***	***β***	
	15.3402(3)		3.52911(2)	18.1236(5)	132.892(2)	
**Atom**		**Cs**	**Pb**	**I1**	**I2**	**I3**
**Position parameter**	***x***	0.7658(4)	1.8423(1)	1.9840(2)	3.7361(5)	3.3772(6)
	y	1.7645(3)	1.7645(3)	1.7645(3)	3.5291(4)	1.7645(5)
	z	8.2343(1)	-2.2158(1)	0.5507(4)	-1.701(2)	-5.227(2)

References

[1] H. K. Mao, J. Xu, P. M. Bell, *J. Geophys. Res.* **1986**, *91*, 4673.

[2] A. Jaffe, Y. Lin, W. L. Mao, H. I. Karunadasa, *J. Am. Chem. Soc.* **2015**, *137*, 1673.

[3] K. A. Adams, S. D. Jacobsen, Z. Liu, S.-M. Thomas, M. Somayazulu, D. M. Jurdy, *Geophys. Res. Lett.* **2012**, *39*.

[4] Van der Pauw, L.J. *Philips Res. Rep.* **1958**,*13*, 1-9

[5] van der Pauw, L.J. *Philips Tech. Rev.***1958**, *20*, 220.

[6] a) A. R. Oganov, C. W. Glass, *J. Chem. Phys.* **2006**, *124*, 15; b) A. O. Lyakhov, A. R. Oganov, M. Valle, *Comput. Phys. Commun.* **2010**, *181*, 1623; c) A. R. Oganov, A. O. Lyakhov, M. Valle, *Acc. Chem. Res.* **2011**, *44*, 227.

[7] a) M. D. Segall, P. J. D. Lindan, M. J. Probert, C. J. Pickard, P. J. Hasnip, S. J. Clark, M. C. Payne, *J. Phys.: Condens. Matter* **2002**, *14*, 2717; b) J. Clark Stewart, D. Segall Matthew, J. Pickard Chris, J. Hasnip Phil, I. J. Probert Matt, K. Refson, C. Payne Mike, in *Z. Kristallogr. - Cryst. Mater.*, Vol. 220, 2005, 567.

[8] a) D. M. Ceperley, B. J. Alder, *Phys. Rev. Lett.* **1980**, *45*, 566; b) J. P. Perdew, A. Zunger, *Phys. Rev. B* **1981**, *23*, 5048.
